# Clinical, Laboratory Characteristics, and Microorganism Infection Status in Neonates With Meningitis in Vietnam: A Cross-Sectional Descriptive Study

**DOI:** 10.1155/ijpe/1852521

**Published:** 2025-11-22

**Authors:** Tho Kieu Anh Pham, Binh Thien Nguyen, Phuong Minh Nguyen, Khai Quang Tran

**Affiliations:** ^1^Department of Physiology, Can Tho University of Medicine and Pharmacy, Can Tho City, Vietnam; ^2^Department of Pediatrics, Can Tho University of Medicine and Pharmacy, Can Tho City, Vietnam

**Keywords:** cerebrospinal fluid, neonatal meningitis, real-time PCR

## Abstract

**Objective:**

This study is aimed at describing the clinical and laboratory characteristics and determining the prevalence of microbial pathogens causing neonatal meningitis detected by cerebrospinal fluid (CSF) real-time polymerase chain reaction (PCR).

**Materials and Methods:**

A cross-sectional descriptive study was conducted on 55 neonates diagnosed with meningitis. Pathogens were identified by using CSF real-time PCR, CSF culture, or blood culture.

**Results:**

Late-onset meningitis accounted for 85.5% (47/55) of cases. The most common clinical signs were respiratory distress (56.4%), fever (47.3%), and lethargy (34.5%). The median white blood cell count was 13,560/mm^3^ (8645–20,709/mm^3^), and the median absolute neutrophil count was 6063/mm^3^ (2895–12,508/mm^3^). C-reactive protein levels were higher in full-term neonates (8.55 mg/L) compared to preterm neonates (6.4 mg/L). The median CSF protein level was 1.4 g/L (1.1–1.73 g/L), with preterm neonates having significantly higher levels (1.59 vs. 1.22 g/L; *p* = 0.013). Blood cultures identified seven cases. Real-time PCR detected pathogens in only three cases (5.5%). No demographic or clinical factors were found to significantly differ between the groups with and without identified microbial pathogens (*p* > 0.05).

**Conclusion:**

Neonatal meningitis commonly manifests with respiratory distress, fever, and lethargy. While real-time PCR enables the detection of viral and rare pathogens, its limited overall detection rate underscores the importance of early diagnosis based on clinical presentation and CSF analysis rather than exclusive reliance on microbiological evidence, which may delay essential treatment.

## 1. Introduction

Meningitis is defined as an infectious and inflammatory process affecting the meninges, the subarachnoid space, and the vascular structures of the brain, and it remains a life-threatening disease [1]. Despite advances in medical care, neonatal meningitis remains a devastating neurological emergency characterized by high rates of mortality and morbidity. Globally, neonatal meningitis affects an estimated 0.3–0.8 per 1000 live births, with mortality rates ranging from 10% to 15% in high-income countries and 40% to 58% in low- and middle-income countries [2]. In South Asia, sub-Saharan Africa, and Latin America, an estimated 1.7 million cases of neonatal sepsis were recorded, of which approximately 200,000 were meningitis, and among the survivors, nearly 23%, equivalent to approximately 18,000 children, have moderate to severe neurodevelopmental disorders [3]. In Canada, the incidence of neonatal meningitis has been reported at 2.2–3.5 per 1000 neonatal intensive care unit admissions, while data from Vietnam in 2020 identified a prevalence of 5.56 cases per 1000 hospitalized neonates, underscoring the substantial disease burden in this population [4, 5].

The diagnosis of meningitis currently depends primarily on cerebrospinal fluid (CSF) analysis and culture, given the variable and nonspecific clinical presentation [6]. Failure to treat or a delay in the treatment might result in complications or prove to be fatal. Although a variety of bacterial species can cause meningitis in neonates, the most commonly identified pathogens in recent years include *Streptococcus pneumoniae*, group B *Streptococcus agalactiae*, *Escherichia coli*, and *Listeria monocytogenes* [7]. While CSF culture is regarded as the gold standard for diagnosing meningitis, its efficacy in newborns is constrained by low sensitivity, extended turnaround times for microbiological results, and a diminished detection rate of pathogens, largely due to the rising number of neonates who have already been administered antibiotics prior to lumbar puncture [5, 8, 9]. Real-time polymerase chain reaction (PCR) has emerged as a promising molecular technique for the swift detection of pathogens, characterized by its high sensitivity and specificity. While it is widely used for detecting pathogens in infections like pneumonia and diarrhea, its efficacy in neonatal meningitis requires further investigation [10, 11].

A thorough comprehension of the microorganisms linked to newborn meningitis, as well as its clinical manifestations and laboratory changes, is crucial for aiding clinicians in executing timely interventions. Despite the fact, there was a limited amount of current information available on the epidemiology of neonatal meningitis in Vietnam. This study is aimed at consolidating the clinical manifestations, laboratory findings, and state of microbial agents in neonates with meningitis at the Can Tho Children's Hospital—the largest pediatric specialty hospital in the Mekong Delta, southern Vietnam, acting as a regional tertiary care center—with two primary objectives: to describe the clinical and laboratory characteristics of neonates with meningitis and to determine the prevalence of microbial pathogens identified through CSF real-time PCR in these neonates. The findings can improve the care and treatment of neonates with meningitis.

## 2. Materials and Methods

### 2.1. Research Ethics

The study protocol was approved by the Institutional Review Board Committee for Ethics Committee in Biomedical Research of Can Tho University of Medicine and Pharmacy, Can Tho City, Vietnam (Approval No. 23.098.HV/PCT-HĐĐĐ dated March 20, 2023).

For all neonatal participants, informed consent was obtained from the parents or legal guardians prior to study enrollment. Parents were provided with comprehensive information regarding the study's objectives, the clinical indication and procedure of lumbar puncture, potential risks, possible benefits, and their right to withdraw their child from the study at any time without affecting the infant's medical care. The procedure and related information were explained in clear, nontechnical language, and parents were given sufficient time to ask questions and consider their decision. Written informed consent explicitly authorizing the lumbar puncture was signed by the parents or legal guardians in accordance with ethical guidelines and institutional policies. As the participants were neonates, assent was not applicable.

### 2.2. Study Design and Setting

The research was designed as a cross-sectional descriptive study and was conducted from March 2023 to February 2025 at the Neonatal Department of Can Tho Children's Hospital, Can Tho City, Vietnam.

### 2.3. Study Population

The source population for this study comprised all neonates admitted to the Neonatal Department of Can Tho Children's Hospital, Can Tho City, Vietnam. The study population included neonates who were diagnosed with meningitis and admitted to the department during the data collection period.

### 2.4. Eligibility Criteria

Eligible participants were neonates aged 0–28 days who met the clinical diagnostic criteria for meningitis, presenting with at least one of the following symptoms: temperature instability, lethargy, drowsiness, hypotonia, seizures or spasms, poor feeding, vomiting, apnea, or cyanosis. Additionally, inclusion required the detection of at least one microbial pathogen through CSF real-time PCR or CSF culture. In cases where both CSF real-time PCR and culture yielded negative results, CSF analysis was used for diagnosis if at least one of the following criteria was met: leukocyte count ≥ 20 cells/mm^3^, protein concentration > 1 g/L, and glucose levels < 30 mg/dL (< 1.7 mmol/L) in term neonates or < 20 mg/dL (< 1.1 mmol/L) in preterm neonates [2, 12, 13]. Participation in the study required mandatory consent from parents or legal guardians. Neonates with congenital or acquired immunodeficiency disorders were excluded from the study.

### 2.5. Sample Collection and Laboratory Procedures

All neonates admitted to the Neonatal Department of Children's Hospital of Can Tho with clinical suspicion of meningitis underwent lumbar puncture to collect CSF samples. The CSF was cultured and analyzed for cell count, protein, and glucose using the AU480 automated biochemistry analyzer (Beckman Coulter Inc., United States) and tested by real-time PCR for 62 pathogens at the Vietnam Research and Development Institute of Clinical Microbiology, Ho Chi Minh City, Vietnam, a laboratory that meets ISO 9001:2015, 13485:2017, and WHO-GMP (see Appendix 1). The laboratory procedure is described as follows:

• The entire CSF sample was first centrifuged to obtain a pellet, which was then lysed using proteinase K. Lysis was performed using the ^NK^PROKPREP kit by adding one-tenth of the kit reagent volume to an equal volume of the sample, sealing tightly, and incubating overnight at 60°C.
• Prior to nucleic acid extraction, sample preparation was performed as follows. A sterile Eppendorf tube was prepared with 100 *μ*L of the negative control sample (−). The number of test samples to be analyzed was counted, and the corresponding number of sterile Eppendorf tubes was prepared. For each tube, 100 *μ*L of the test sample lysed with proteinase K was added. The ^NK^MTBDNA-IC tube was vortexed vigorously three times for 10 s each, and 10 *μ*L of ^NK^MTBDNA-IC was then added into each negative control tube and each test sample tube using a micropipette. All negative control and test sample tubes were subsequently vortexed to ensure thorough mixing.
• Nucleic acid extraction was performed using the ZiXpress 32 system (Zinexts Life Science Corp., Taiwan) with the ^NK^DNARNAprep-MAGBEAD 46 kit (Nam Khoa Company, Vietnam). This kit was validated against the BOOM method for nucleic acid extraction. After extraction, the DNA was eluted in 50 *μ*L of 1X TE buffer for PCR preparation.
• The extracted nucleic acids were then subjected to real-time PCR using the CFX96 Touch system (Bio-Rad Laboratories, United States) with specific primers and TaqMan probes to detect and quantify the pathogens.

A real-time PCR result was provided within 24 h. A positive real-time PCR was defined as the detection of a pathogen at > 10^5^ copies/mL.

For each eligible neonate, data were collected on demographic characteristics (age, sex, birth weight, gestational age, and disease onset timing), clinical features (e.g., presenting symptoms, e.g., fever, respiratory distress, lethargy, poor feeding, jaundice, abdominal distension, vomiting, cyanosis, seizures, diarrhea, and umbilical infection), and laboratory findings. Laboratory investigations comprised hematological parameters, including hematocrit (Hct), hemoglobin (Hb), white blood cell count (WBC), absolute neutrophil count (ANC), and platelet count, which were performed using the SIEMENS ADVIA 2120i hematology analyzer (Siemens Healthineers, Germany). C-reactive protein (CRP) was measured by the AU480 biochemical analyzer (Beckman Coulter Inc., United States). Microbiological investigations included CSF and blood cultures performed with the BACT/ALERT 3D system, certified under ISO 9001:2000, ANSI/ISO/ASQ9001:2000, and ISO 13458:2003 quality standards, as well as CSF characteristics and PCR results.

### 2.6. Operational Definitions

Operational definitions of study variables are provided in Appendix 2.

### 2.7. Statistical Analysis

Data were analyzed using standard medical statistical methods and entered into the Statistical Package for Social Sciences (SPSS) Version 26.0 (IBM, Armonk, New York, United States). Frequencies and percentages were calculated for categorical variables, while means and standard deviations (or medians and interquartile ranges [IQRs]) were determined for continuous variables. Differences between proportions were compared using the *χ*^2^ test or Fisher's exact test, and differences in means/medians between groups were assessed using the independent samples *T*-test (for normally distributed data) or Mann–Whitney *U* test (for nonnormally distributed data). A *p* value < 0.05 was considered statistically significant.

## 3. Results

### 3.1. Demographic Characteristics

From March 2023 to February 2025, 55 neonates with meningitis were admitted to the Neonatal Department of Children's Hospital of Can Tho.  Table 1 describes the demographic characteristics of participants. The median age at admission was 5 days (IQR 1–14.5). Males predominated (*n* = 32, 58.2%). The median gestational age was 37 weeks (IQR 33.5–38), with 38.2% (*n* = 21) born preterm and 61.8% (*n* = 34) at term. A total of 12.7% (*n* = 7) were classified as very low birth weight, 25.5% (*n* = 14) as low birth weight, and 61.8% (*n* = 34) as normal birth weight. The majority of cases (85.5%, *n* = 47) had late-onset meningitis (Table 1).

### 3.2. Clinical and Laboratory Characteristics

The predominant clinical manifestations in neonates with meningitis included respiratory distress (56.4%), fever (47.3%), and lethargy (34.5%). Respiratory distress was substantially more frequent in preterm compared with term neonates (85.7% vs. 38.2%), corresponding to a markedly elevated odds ratio (OR = 9.69, 95% CI: 2.38–39.48, *p* < 0.001). Conversely, fever and poor feeding occurred predominantly among term infants (61.8% vs. 23.8% and 50.0% vs. 4.8%, respectively), both associated with significantly lower odds in the preterm group (OR = 0.08, 95% CI: 0.02–0.33, *p* < 0.001; OR = 0.05, 95% CI: 0.01–0.42, *p* < 0.001).

Laboratory analysis demonstrated no statistically significant differences in hematological parameters—including WBC, ANC, Hb, Hct, and platelet count—with small effect sizes suggesting minimal clinical relevance. CRP was elevated (≥ 1 mg/dL) in 20 episodes (36.4%). Median CRP levels were higher in term neonates (8.55 mg/L, IQR 3.0–27.3) compared to preterm neonates (6.4 mg/L, IQR 3.26–14.2); however, this difference did not achieve statistical significance (*p* = 0.249).

Among CSF parameters, protein concentration was significantly higher in preterm neonates (median 1.59 g/L, IQR 1.12–6.37) than in term neonates (median 1.22 g/L, IQR 0.62–3.67; *p* = 0.013). The effect size was small to moderate (*r* = 0.39, 95% CI: 0.11–0.63), indicating a clinically meaningful difference. In contrast, CSF leukocyte counts, glucose levels, and CSF-to-blood glucose ratios did not differ significantly between groups (all *p* > 0.05), with negligible effect sizes (Table 2).

### 3.3. Microbial Pathogen Characteristics

Blood culture identified pathogens in seven cases, representing six different organisms. *Staphylococcus* species predominated with four isolates (*S. hominis* ssp. *hominis*, *S. aureus*, and *S. epidermidis*), while *Acinetobacter* spp., *Candida albicans*, and *Enterococcus faecium* each accounted for one case. Real-time PCR of CSF detected pathogens in three cases, including one bacterial pathogen (*Salmonella* sp.) and two viral pathogens, Human Herpesvirus 6 (HHV-6) and cytomegalovirus. No pathogens were recovered by CSF culture, and there was no overlap between organisms detected by blood culture and those identified by real-time PCR (Table 3).

Among the 55 neonates included in this study, microbial pathogens were identified in nine cases, with nine distinct pathogens detected through a total of 10 clinical samples, including blood and CSF. One case tested positive in both blood culture and CSF real-time PCR. No significant differences were observed in demographic or clinical characteristics between neonates with detected pathogens and those in whom no causative pathogen was identified (Table 4).

## 4. Discussion

In this study, the most frequently observed clinical manifestations of neonatal meningitis were respiratory distress, fever, and lethargy, reflecting the nonspecific nature of the disease at presentation. Notably, respiratory distress was more frequently observed in preterm neonates, whereas fever and poor feeding predominated among term neonates, suggesting gestational age–related differences in presentation. Despite comprehensive CSF microbiological testing using both culture and PCR, pathogens were identified in only three (5.5%) cases.

This study found that neonatal meningitis predominantly presents as late-onset meningitis. This finding aligns with the 7-year retrospective study by Biset and colleagues, which reported a late-onset meningitis frequency of 89.4% [14]. A Canadian study similarly reported that late-onset meningitis accounted for up to 87% of cases [4]. Another study by Gaschignard et al. of a cohort of 444 neonatal meningitis cases over 7 years noted that late-onset cases were more common than early-onset cases (68% vs. 32%) [15]. The lack of a standardized definition for early versus late-onset neonatal infections has led some studies to use a threshold of 3 days, while others use 7 days. Improved prenatal care practices (e.g., Maternal Group B *Streptococcus* screening and intrapartum antibiotic prophylaxis) have contributed to the declining incidence of early-onset sepsis. This study observed a male predominance, with a male-to-female ratio of 1.4:1. This finding is consistent with the results reported by Nga Nguyen et al., who documented a ratio of 1.1:1 [5]. Another study reported that 72.73% of infants diagnosed with meningitis were male [1]. Kelly et al. reported higher morbidity and mortality in male neonates linked to sex hormones, X-linked immunity, and inflammatory differences [16].

The clinical features of neonatal meningitis are inherently nonspecific, and some infants in our cohort were categorized as having clinical meningitis despite negative CSF culture and PCR results. This approach reflects common clinical practice, in which diagnosis relies on a combination of compatible symptoms and abnormal CSF indices when microbiological confirmation is lacking. Furthermore, it is possible that a proportion of these infants had systemic sepsis with neurological involvement rather than true meningitis, a distinction that remains challenging in neonatal populations. Our findings on common clinical features are consistent with a cross-sectional study from Iran by Boskabadi et al. involving 468 neonates with suspected infection. The authors reported that respiratory symptoms were the most prevalent clinical signs in newborns with meningitis, followed by general and gastrointestinal manifestations [17]. This respiratory condition, which may stem from an underlying illness or neonatal respiratory problems, often necessitates frequent medical interventions, thereby increasing the risk of infection. Clinical presentation also differed according to gestational age. Preterm neonates most frequently manifested respiratory distress, whereas term neonates more often presented with fever and poor feeding. This gestational age–related pattern is concordant with prior evidence. Shekhar and Merchant noted that the predominant signs in term infants are fever, lethargy, and poor feeding, while preterm infants chiefly demonstrate respiratory decompensation [18].

Existing evidence suggests that peripheral WBC count alone is inadequate for guiding lumbar puncture decisions [19, 20]. Consistent with prior studies, our findings demonstrate that WBC counts and CRP levels at the onset of meningitis lack sufficient diagnostic utility, as the recorded values were all within normal ranges. In neonates, CSF protein concentrations exceeding the diagnostic threshold of ≥ 1 g/L and leukocyte counts of ≥ 20 cells/mm^3^ are strongly associated with an increased likelihood of meningitis [13]. This pattern is consistent with our study. By contrast, CSF glucose concentrations in our cohort were higher than the conventional threshold for meningitis, underscoring the limited discriminatory value of glucose indices in this age group. Consistent with previous studies, CSF glucose alone is a poor predictor of neonatal meningitis and is influenced by confounders such as antibiotic pretreatment [13, 21]. Moreover, the CSF-to-serum glucose ratio offers little diagnostic utility in acutely ill neonates, as serum glucose may be transiently elevated due to physiological stress or intravenous glucose administration before sampling.

The pathogen yield from CSF was low. No organisms were recovered by CSF culture, and real-time PCR identified pathogens in only three cases (5.5%). These findings are consistent with prior reports, with Garges et al. reporting CSF culture positivity of 1% among 9111 neonates with meningitis and Buttera et al. identifying pathogens by CSF PCR in 7.5% of a cohort of 40 neonates [19, 20]. Several factors likely contributed to the low diagnostic yield. Empiric antibiotics are frequently initiated immediately in suspected neonatal sepsis or meningitis. Even a single dose can rapidly sterilize the CSF, which reduces culture positivity [22]. Thus, a negative CSF result does not exclude meningitis if lumbar puncture is followed by antibiotics. In addition, targeted molecular assays are constrained by their predefined analyte lists. Pathogens absent from the panel, or with sequence variation preventing primer or probe binding, will go undetected. This constraint introduces a relevant risk of false-negative results [23].

This study has several limitations, including a small sample size and a cross-sectional design. Future studies with larger cohorts and longitudinal follow-up are necessary to gain a more comprehensive understanding of neonatal meningitis.

## 5. Conclusion

Neonatal meningitis is often characterized by late onset, with respiratory distress, fever, and lethargy being the most common symptoms. However, the low detection rate of microbial pathogens highlights the necessity of a comprehensive diagnostic approach that combines clinical evaluation and laboratory findings. Early diagnosis based on clinical symptoms and CSF analysis is essential to minimize dependence on microbiological results, which have a relatively low detection rate. In summary, findings from this study could inform national neonatal meningitis diagnostic algorithms, improve timely pathogen identification, and guide empirical treatment strategies in Vietnam.

## Figures and Tables

**Table 1:** Demographic characteristics of the study population (*n* = 55).

**Characteristic**	**Frequency**	**Percentage (%)**
Age at admission (days)^a^	5 (1–14.5)	
Sex		
Male	32	58.2
Female	23	41.8
Gestational age (weeks)^b^	37 (33.5–38)	
28–< 32 weeks	7	12.7
32–< 37 weeks	14	25.5
≥ 37 weeks	34	61.8
Birth weight (grams)^b^	2688.7 ± 918.1	
< 1500 g	7	12.7
1500–2499 g	14	25.5
≥ 2500 g	34	61.8
Timing of disease onset (days)^b^	13.7 ± 7.9	
< 3 days	8	14.5
≥ 3 days	47	85.5

^a^Values expressed as median (IQR).

^b^Values expressed as mean ± standard deviation.

**Table 2:** Clinical features, laboratory parameters, and cerebrospinal fluid analysis in neonates with meningitis (*n* = 55).

**Parameter/clinical signs**	**Total value**	**Term value (** **n** = 34**)**	**Preterm value (** **n** = 21**)**	**OR (95% CI)**	**p** ** value**
Clinical signs					
Respiratory distress	31 (56.4)	13 (38.2)	18 (85.7)	9.69 (2.38–39.48)	**< 0.001**
Fever	26 (47.3)	21 (61.8)	5 (23.8)	0.08 (0.02–0.33)	**< 0.001**
Lethargy	19 (34.5)	13 (38.2)	6 (28.6)	0.65 (0.2–2.09)	0.464
Poor feeding	18 (32.7)	17 (50.0)	1 (4.8)	0.05 (0.01–0.42)	**< 0.001**
Jaundice	17 (30.9)	8 (23.5)	9 (42.9)	2.44 (0.76–7.88)	0.132
Abdominal distension	13 (23.7)	6 (17.6)	7 (33.3)	2.33 (0.66–8.27)	0.208⁣^∗^
Vomiting	6 (10.9)	6 (17.6)	0 (0)		0.072⁣^∗^
Cyanosis	6 (10.9)	2 (5.9)	4 (19)	3.77 (0.63–22.69)	0.188⁣^∗^
Seizures	5 (9.1)	4 (11.8)	1 (4.8)	0.38 (0.04–3.61)	0.639⁣^∗^
Diarrhea	3 (5.4)	1 (2.9)	2 (9.5)	3.47 (0.29–40.9)	0.551⁣^∗^
Umbilical infection	3 (5.4)	1 (2.9)	2 (9.5)	3.47 (0.29–40.9)	0.551⁣^∗^
Laboratory findings					
WBC (/mm^3^)^a^	13,560 (8645–20,709)	13,385 (8370–21,010)	14,230 (9640–18,850)	0.02 (0.002–0.31)	0.903⁣^∗∗^
ANC (/mm^3^)^a^	6063 (2895–12,508)	6454 (2746–12,747)	4989 (3474–12,294)	0.03 (0.005–0.31)	0.835⁣^∗∗^
Hb (g/dL)^b^	13.465 ± 2.763	13.482 ± 2.736	13.438 ± 2.874	0.02 (−0.53–0.56)	0.955
Hct (%)^b^	41.64 ± 8.722	42.262 ± 8.524	40.633 ± 9.154	0.19 (−0.36–0.73)	0.506
Platelets (/mm^3^)^b^	311,510 ± 152,190	338,706 ± 152,516	267,477 ± 144,438	0.48 (−0.08–1.03)	0.092
CRP (mg/L)^a^	7.8 (3.13–17.04)	8.55 (3–27.3)	6.4 (3.26–14.2)	0.16 (0.007–0.4)	0.249⁣^∗∗^
CSF parameters^a^					
Protein (g/L)^c^	1.4 (1.1–1.73)	1.22 (0.89–1.59)	1.59 (1.3–1.9)	0.39 (0.1–0.63)	**0.013 **⁣^∗∗^
Leukocytes (cells/mm^3^)	20 (10–60)	20 (10–70)	20 (10–50)	0.1 (0.006–0.36)	0.467⁣^∗∗^
Glucose (mmol/L)	3.1 (2.4–3.7)	3.05 (2.4–3.4)	3.2 (2.3–3.8)	0.06 (0.006–0.34)	0.677⁣^∗∗^
CSF glucose/blood glucose	0.68 (0.56–0.95)	0.65 (0.57–1.07)	0.78 (0.5–0.93)	0.03 (0.006–0.33)	0.802⁣^∗∗^

*Note: *Statistically significant values (*p* < 0.05) are indicated in boldface.

Abbreviations: ANC, absolute neutrophil count; CRP, C-reactive protein; CSF, cerebrospinal fluid; Hb, hemoglobin; Hct, hematocrit; WBC, white blood cell count.

^a^Values expressed as no. (%) or median (IQR).

^b^Values expressed as mean ± standard deviation.

^c^ *n* = 41: Due to reagent depletion during the sampling process, the test could not be performed.

⁣^∗^Fisher's exact test; ⁣^∗∗^Mann–Whitney *U* test.

**Table 3:** Identification of microbial pathogens by blood culture and cerebrospinal fluid real-time polymerase chain reaction.

**Pathogen**	**Blood culture**	**CSF real-time PCR**
*Enterococcus faecium*	1	0
*Staphylococcus hominis* ssp. *hominis*	2	0
*Staphylococcus aureus*	1	0
*Staphylococcus epidermidis*	1	0
*Acinetobacter* spp.	1	0
*Candida albicans*	1	0
HHV-6	0	1
*Salmonella* sp.	0	1
*Cytomegalovirus*	0	1
Total	7	3

Abbreviations: CSF, cerebrospinal fluid; PCR, polymerase chain reaction.

**Table 4:** Association between demographic characteristics, clinical signs, and pathogen detection (*n* = 55).

**Demographic characteristics/clinical signs**		**Pathogen (+) ** **n** ** (%) (** **n** = 9**)**	**Pathogen (−) ** **n** ** (%) (** **n** = 46**)**	**OR (95% CI)**	**p** ** value**
Sex	Male	7 (77.8)	25 (54.3)	2.94 (0.55–15.69)	0.277⁣^∗^
	Female	2 (22.2)	21 (45.7)		
Gestational age	< 37 weeks	5 (55.6)	16 (34.8)	0.43 (0.1–1.82)	0.279⁣^∗^
	≥ 37 weeks	4 (44.4)	30 (65.2)		
Birth weight	< 2500 g	5 (55.6)	16 (34.8)	0.43 (0.1–1.82)	0.279⁣^∗^
	≥ 2500 g	4 (44.4)	30 (65.2)		
Timing of disease onset	< 3 days	1 (11.1)	7 (15.2)	0.69 (0.08–6.47)	1⁣^∗^
	≥ 3 days	8 (88.9)	39 (84.8)		
Respiratory distress	Yes	5 (55.6)	26 (56.5)	1.04 (0.25–4.38)	1⁣^∗^
	No	4 (44.4)	20 (43.5)		
Fever	Yes	5 (55.6)	21 (45.7)	0.67 (0.16–2.83)	0.721⁣^∗^
	No	4 (44.5)	25 (54.3)		
Lethargy	Yes	3 (33.3)	16 (34.8)	1.07 (0.24–4.84)	1⁣^∗^
	No	6 (66.7)	30 (65.2)		
Poor feeding	Yes	4 (44.4)	14 (30.4)	0.55 (0.13–2.35)	0.454⁣^∗^
	No	5 (55.6)	32 (69.6)		
Jaundice	Yes	3 (33.3)	14 (30.4)	0.88 (0.19–4)	1⁣^∗^
	No	6 (66.7)	32 (69.6)		
Abdominal distension	Yes	3 (33.3)	10 (21.7)	0.56 (0.12–2.63)	0.428⁣^∗^
	No	6 (66.7)	36 (78.3)		
Vomiting	Yes	0 (0)	6 (13)		0.574⁣^∗^
	No	9 (100)	40 (87)		
Cyanosis	Yes	1 (11.1)	5 (10.9)	0.98 (0.1–9.51)	1⁣^∗^
	No	8 (88.9)	41 (89.1)		
Seizures	Yes	0 (0)	5 (10.9)		0.578⁣^∗^
	No	9 (100)	41 (89.1)		
Diarrhea	Yes	1 (11.1)	2 (4.3)	0.36 (0.03–4.5)	0.421⁣^∗^
	No	8 (88.9)	44 (95.7)		
Umbilical infection	Yes	1 (11.1)	2 (4.3)	0.36 (0.03–4.5)	0.421⁣^∗^
	No	8 (88.9)	44 (95.7)		

⁣^∗^Fisher's exact test.

**Table 5:** Microbiological pathogens detected by real-time polymerase chain reaction.

**Bacterial agents**
*Neisseria meningitidis*
*Streptococcus pneumoniae*
*Streptococcus suis*
*Streptococcus pyogenes* (GAS)
*Streptococcus agalactiae* (GBS)
*Haemophilus influenzae* Type B
*Salmonella* sp.
*Elizabethkingia meningoseptica*
*Listeria monocytogenes*
*Escherichia coli*
*Escherichia coli* K1
*Brucella*
*Treponema pallidum*
*Leptospira interrogans*
*Rickettsia prowazekii*
*Orientia tsutsugamushi*
*Actinomyces israelii*
**Mycobacterium**
*Mycobacterium tuberculosis*
*Nocardia asteroides*
Nontuberculosis (NTM)
**Parasites**
*Acanthamoeba*
*Naegleria fowleri*
*Balamuthia mandrillaris*
*Entamoeba histolytica*
*Baylisascaris procyonis*
*Toxoplasma gondii*
*Angiostrongylus cantonensis*
*Strongyloides stercoralis*
*Taenia solium*
*Gnathostoma spinigerum*
**Viral Agents**
Herpes Simplex Virus Type 1 (HSV-1)
Herpes Simplex Virus Type 2 (HSV-2)
Lymphocytic choriomeningitis GP
Lymphocytic choriomeningitis NP2
Cytomegalovirus (CMV)
Varicella–Zoster virus (VZV)
Epstein–Barr virus (EBV)
Human Herpesvirus 6 (HHV-6)
Enterovirus 71 (EV-71)
Echovirus/Coxsackievirus A, B (EV)
Saffold virus
Human parechovirus
Japanese B. encephalitis (JBE)
Influenza Virus A
Measles virus
Rubella virus
Rabies virus
Zika virus
Mumps virus (MuV)
Parvovirus B19
John Cunningham virus (JCV)
BK virus (BKV)
Adenovirus
Dengue virus
**Fungal agents**
*Cryptococcus neoformans*
*Pneumocystis carinii*
*Penicillium marneffei*
*Coccidioides immitis/posadasii*
*Scotoplanes globosa*
*Sporothrix schenckii/brasiliensis*
*Mucormycosis*
*Candida glabrata*

**Table 6:** Operational definitions used in the study.

**Category**	**Definition**
Term neonates	Neonates with gestational age ≥ 37 weeks [14]
Very preterm neonates	Neonates with gestational age 28 to less than 32 weeks [14]
Moderate to late preterm neonates	Neonates with gestational age 32 to less than 37 weeks [14]
Very low birth weight	Birth weight less than 1500 g [14]
Low birth weight	Birth weight 1500 to 2499 g [14]
Normal birth weight	Birth weight of 2500 g or greater [14]
Early- and late-onset meningitis	Presence of clinical signs of meningitis and isolation of the pathogen from CSF within or after 72 h, respectively [15]
Fever	Rectal temperature greater than 38°C [16]
Respiratory distress	Silverman Andersen respiratory severity score greater than or equal to 3 [17]
Poor feeding	Ineffective sucking or breastfeeding fewer than eight times within 24 h [18]
Diarrhea	An increase in stool frequency accompanied by decreased stool consistency compared with the normal pattern [19]

## Data Availability

The data that support the findings of this study are available on request from the corresponding author. The data are not publicly available due to privacy or ethical restrictions.
